# Supplementary material for: Analysis of chi angle distributions in free amino acids via multiplet fitting of proton scalar couplings
Source: Magn Reson (Gott). 2024 Aug 19;5(2):103–20. doi: 10.5194/mr-5-103-2024 (PMC11570886; doi:10.5194/mr-5-103-2024)
Supplement: The supplement related to this article is available online at: https://doi.org/10.5194/mr-5-103-2024-supplement. [file mr-5-103-2024-supplement.zip › Syed2024/data/fit1d_fitnmr_output.tar.gz!/fit1d/ala/400/bmse000028_simulation_1_nmredata/1H/resonances_1d.pdf]

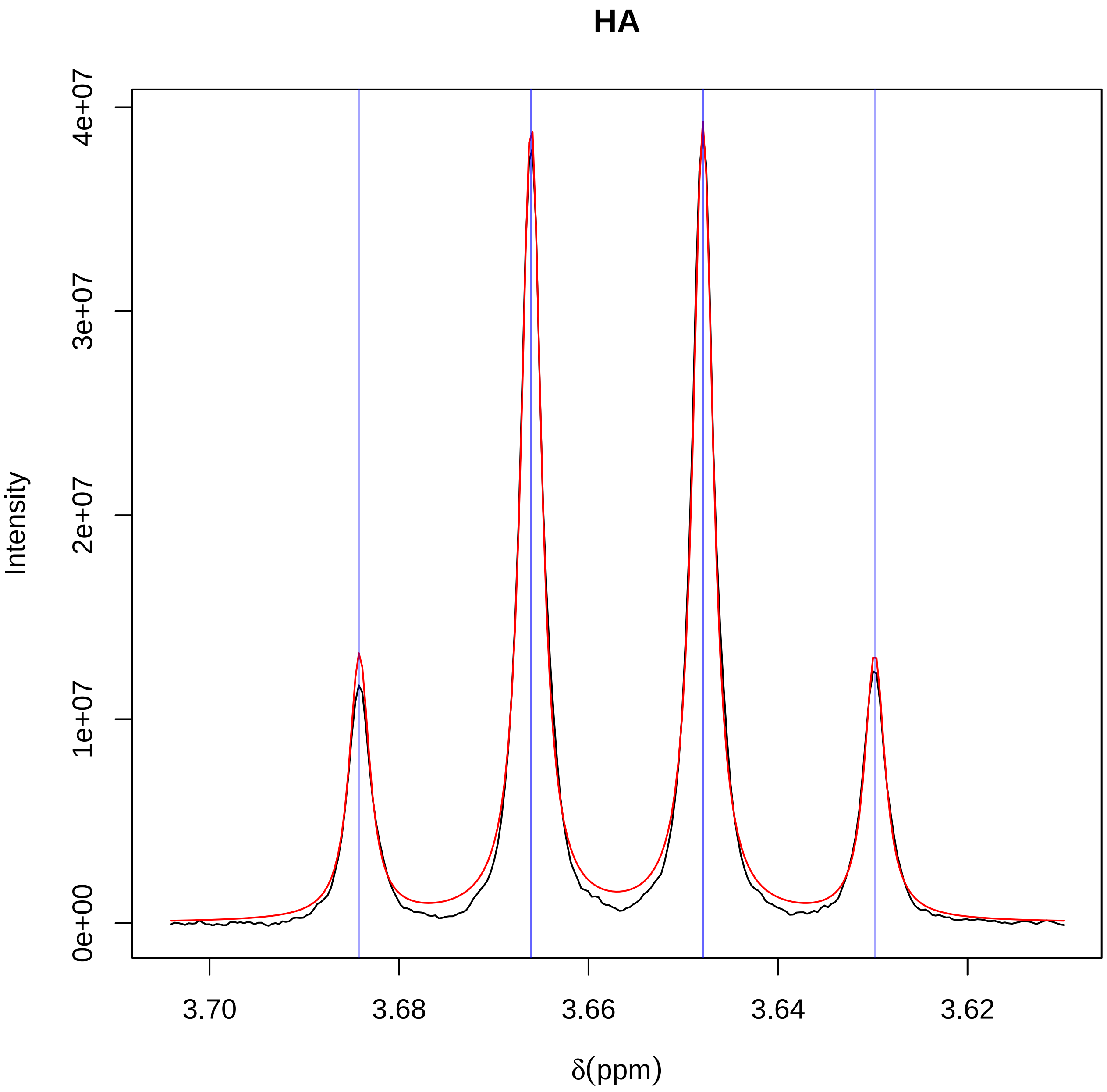

HA
Intensity
4e+07
3e+07
2e+07
1e+07
0e+00
3.70
3.68
3.66
3.64
3.62
δ(ppm)

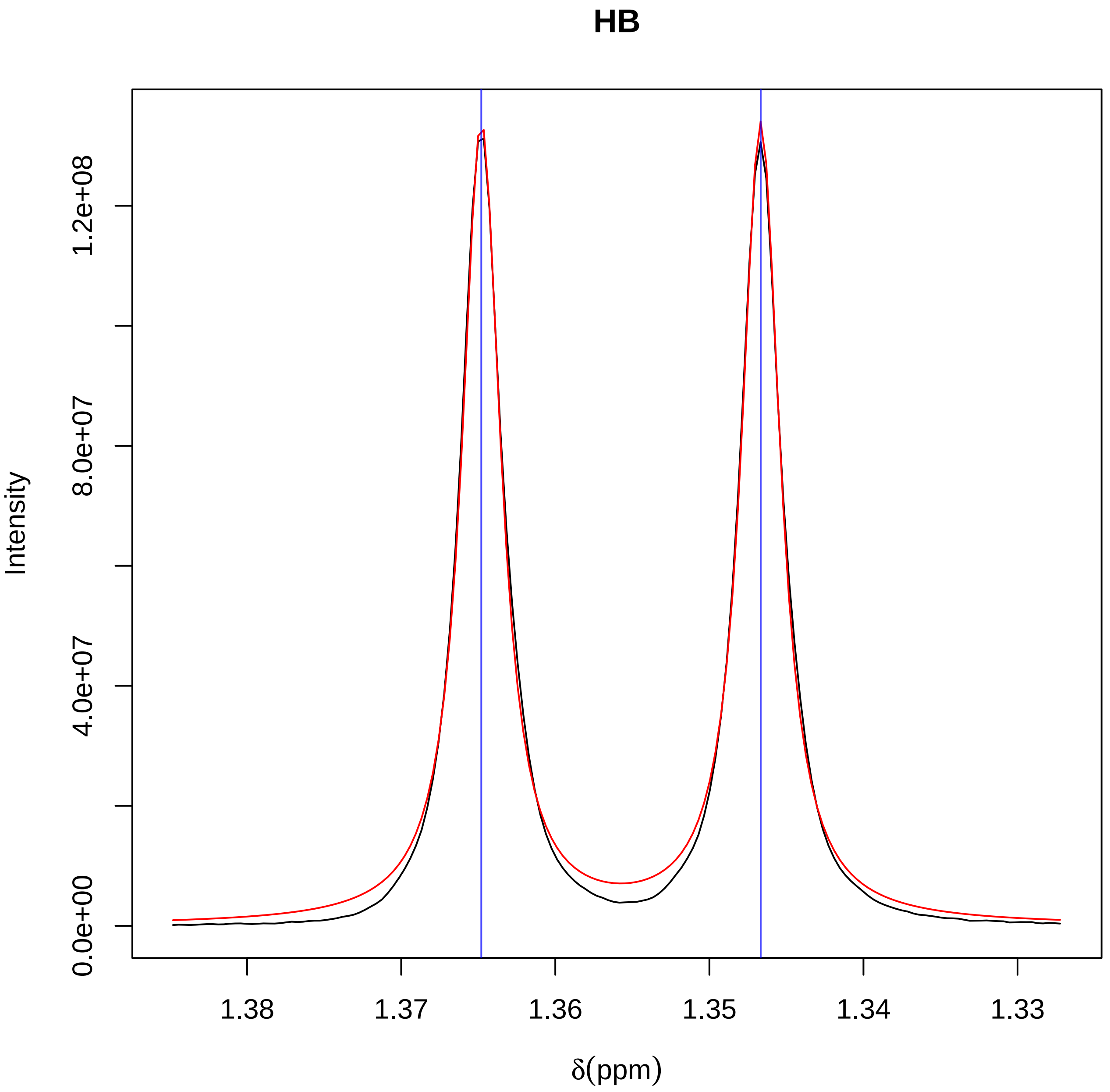
HB
Intensity
0.0e+00
4.0e+07
8.0e+07
1.2e+08
1.38
1.37
1.36
1.35
1.34
1.33
δ(ppm)
